# Feasibility of gamified visual desensitisation for visually-induced dizziness

**DOI:** 10.1038/s41598-024-67745-9

**Published:** 2024-08-01

**Authors:** Nathan Goodwin, Georgina Powell, Fernando Loizides, Hannah Derry-Sumner, Deepak Rajenderkumar, Petroc Sumner

**Affiliations:** 1 School of Psychology, Cardiff University, Cardiff, UK; 2 School of Computer Science and Informatics, Cardiff University, Cardiff, UK; 3 University Hospital of Wales, Cardiff, UK

**Keywords:** Visually-induced dizziness, Visual vertigo, Vestibular rehabilitation, Virtual environment, VR, Functional dizziness, Central dizziness, Psychology, Health care, Medical research, Neurology

## Abstract

Visually-induced dizziness (visual vertigo) is a core symptom of Persistent Perceptual Postural Dizziness (PPPD) and occurs in other conditions and general populations. It is difficult to treat and lacks new treatments and research. We incorporated the existing rehabilitation approach of visual desensitisation into an online game environment to enhance control over visual motion and complexity. We report a mixed-methods feasibility trial assessing: Usage and adherence; rehabilitation potential; system usability and enjoyment; relationship with daily dizziness. Participants played online with (intervention, N = 37) or without (control, N = 39) the visual desensitisation component for up to 5–10 min, twice daily for 6 weeks. Dropout was 45%. In the intervention group, N = 17 played for the recommended time while N = 20 played less. Decreases in visual vertigo symptoms, anxiety and depression correlated with playtime for the intervention but not control. System usability was high. Daily symptoms predicted playtime. Qualitative responses broadly supported the gamified approach. The data suggest gamified visual desensitisation is accessible, acceptable and, if adherence challenges can be overcome, could become a useful addition to rehabilitation schedules for visually-induced dizziness and associated anxiety. Further trials are needed.

## Introduction

Visually-induced dizziness, or ‘visual vertigo’, is a debilitating symptom occurring across several disorders and conditions, such as Migraine and Meniere’s Disease, or after Traumatic Brain Injury, and it is a core feature of Persistent Postural Perceptual Dizziness (PPPD), the leading cause of chronic, functional dizziness^1^. It also exists on a spectrum in the healthy population^2^. Patients experience symptoms of dizziness, unsteadiness and non-spinning vertigo that are triggered or exacerbated by visual motion and complex visual environments^1^. Such vulnerability to visual environments tends to be persistent and very difficult to treat. Anxiety is a common correlate, with patients often developing fear of everyday situations that may trigger symptoms^3^.

Current treatment involves daily vestibular rehabilitation exercises and visual desensitisation, aiming to recalibrate sensory integration and reduce hyper-reactivity to visual stimulation^4^. For example, watching recorded optokinetic stimuli (moving bars or light spots) for up to 45 min daily for 8 weeks was found to improve dizziness, posture and gait^5,6^. Clinically, people with visually-induced dizziness are often advised to view videos with radial optic flow or with moving patterned stimuli with the aim to desensitise to these visual inputs relative to information from their vestibular system^7^. Given the common association with anxiety, treatment can also include psychological therapies (e.g. cognitive behavioural therapy, CBT^8^) and pharmacological agents (e.g. selective serotonin re-uptake inhibitors) to break the perpetuating anxiety-dizziness cycle and help patients cope with symptoms in everyday life^4^.

However treatment success is highly variable, and a major challenge for all chronic dizziness rehabilitation is adherence^7^—therapy provokes symptoms and is unengaging^9^. A second limitation is insufficient flexibility for individual patients, who show a wide range of symptom severities and situational triggers for dizziness and anxiety^7^. Too much stimulation too soon inevitably results in discontinuation^10^.

**Figure 1:**
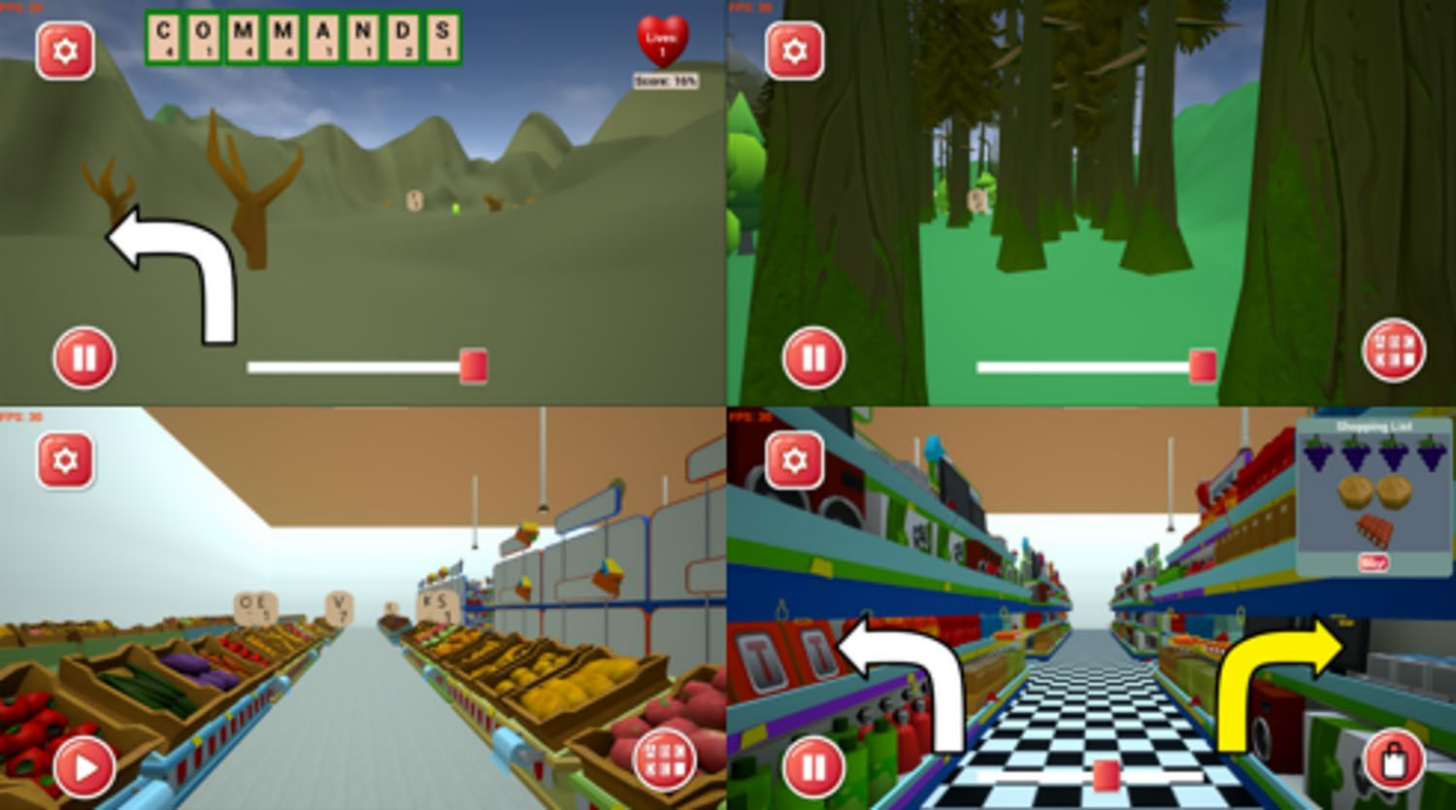
Images from Balance-Land. Players move through virtual environments collecting letters for word games or collecting items from a shopping list. Different zones provide different intensities of visual motion stimulation: the Desert zone (top left) is low contrast and spatial frequency, with a limited colour palette and few objects; the Park zone (top right) steps up these characteristics, with high contrast tree trunks; the Supermarket zone (bottom) has high contrasts and spatial frequencies, with many cluttered objects (supermarkets are a major dizziness trigger for patients^13,14^).

Gamification has helped rehabilitation in other domains, including chronic disease management, physical activity, nutrition, mental health, and hygiene^11^. However, online videos or games containing optic flow potentially suitable for visual desensitisation tend to contain high levels of motion and visual complexity that are too intense for patients with visually-induced dizziness. We therefore developed a new online rehabilitation game (‘*Balance-Land*’) as a puzzle game within an environment where the optic flow and scene complexity can be graded and controlled separately from puzzle difficulty (Fig. 1). Participants are able to choose the environment (Desert, Park, or Supermarket) they feel appropriate for their symptoms, and also adjust motion speed to scale symptom provocation. We have developed the tool through iterative consultation with patients and clinicians^12^, to ensure that it is user-focused and can be tailored to individual patient needs. Balance-Land is free to use and can be accessed and viewed here: https://cudizzylab.org/guide-to-balanceland/. The aim is not to replace other kinds of rehabilitation therapy, but rather to provide an additional pragmatic home-based option for flexible multi-faceted treatment for the range of patients experiencing visually-induced dizziness.

In this paper, we present the results of a semi-randomised mixed-methods 6-week feasibility trial of Balance-Land in which participants played the puzzle games with (intervention group) or without (control group) moving through the virtual environments (the visual desensitisation component). The goals of the feasibility trial were to assess:

1. Dropout and adherence: would participants be willing to use the game twice daily for 5–10 min for 6 weeks?
2. Rehabilitation potential: primary outcome of self-reported visually-induced dizziness symptoms (the visual vertigo analogue scale, VVAS) before and after using the game for 6 weeks and whether this depended on playtime, and secondary outcomes of anxiety, depression and other dizziness questionnaires.
3. System Usability and enjoyment: participants reported useability and previous digital experience, and game data was recorded to assess if all controls and game areas were utilised. They also rated enjoyment.
4. Daily symptoms: participants reported how dizzy or unwell they felt at each game session, so we could assess if this predicted usage.

## Results

### Enrolment, dropout and adherence

**Figure 2:**
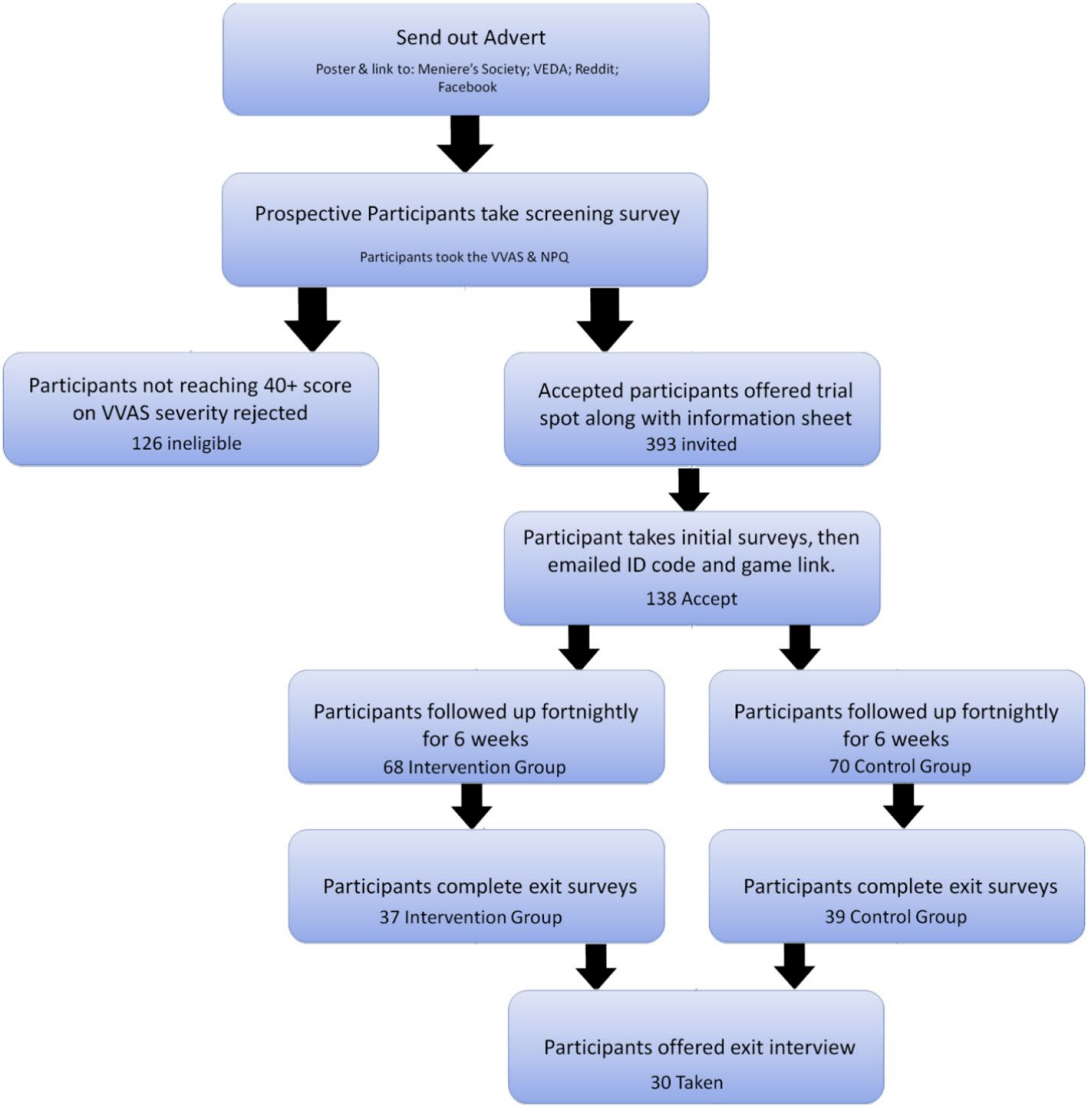
Recruitment and retention pipeline for participants. The enrolment rate was 35% and the retention rate was 55%.

Numbers of participants recruited and completing are given in Fig. 2. The enrolment rate was 35% and the retention rate was 55%. Participants were recruited globally, with the majority from the USA, UK, and Canada. The most common reported current diagnoses were PPPD (37), vestibular migraine (31), and Meniere’s Disease (10) (no significant difference between groups; numbers given are for participants completing the study; see Supplementary Table S1 for more information). These were non-exclusive and many other comorbid conditions were reported. Note that diagnosis for dizziness is challenging and known to be often incorrect (with over-diagnosis of Meniere’s Disease, for example:^15,16^). Hence, we took a symptom-based approach, matching groups for VVAS severity rather than reported diagnoses. The proportion with PPPD was much higher in those who enrolled compared to those invited (meeting inclusion criteria) who did not enrol, but there were no other major differences in characteristics measured at screening (see Supplementary Table S2). Compared to cohorts in the literature with PPPD, vestibular migraine and Meniere’s Disease^17–23^, our enrolled cohort had similar mean age, higher female:male ratio, and higher scores on DHI and HADS (which is to be expected given these correlate with VVAS, where we had an inclusion criterion of > 40; see Supplementary Table S3).

There was no significant difference in dropout rates between intervention and control groups (44% vs 46%, *x*^2^(1,61) = 0.004). Most participants withdrawing from the study did not give a reason. Of those that did, the reasons were: other health issues (6); technical issues (3); time commitment for study too large (2); evoked symptoms too severe (2); difficult daily life (1); game too difficult (1); no effect noticed (1). There was no difference in initial VVAS scores for those that completed vs those that did not (72 vs 70, *t*(136) = 0.94). Neither was there any difference in reported digital experience (*x*^2^(4,137) = 1.5); 64 of 113 (57%) everyday computer users completed, while 13 of 25 (52%) less frequent users completed.

Of the participants who completed the study, 17 adhered to the recommendation of playing, on average, 5–10 min twice daily for 6 weeks (7 h or more in total over 42 days). Twenty participants played less than this (see Table 1 for comparison between these groups). In order to answer the remaining feasibility objectives, it is therefore essential to take playtime into account when assessing the study results (we present correlations with playtime below, and in supplementary information we provide separate results for those adhering to recommendations). Note that any analysis approach utilising playtime breaks the randomisation, because amount of playtime was self-selected by participants.

**Table 1:** Participant information prior to study (at time 1) for all those that completed time 1 assessment (first two columns) and for those that completed time 2, 6 weeks later (right hand four columns; also comparing those that adhered to recommended playtime with those that did not). Means and SD are given, except where data are categorical. Pseudo randomisation aimed to minimise differences in VVAS severity, age and symptom duration across groups (bold rows).

	Participants at Time 1 (scores are time 1)		Participants at Time 2 (scores are from time 1)			
	Control N = 70	Intervention N = 68	Control N = 39	Intervention = 37	Low Playtime N = 20	Recommended N = 17
**VVAS Severity**	**70.2 (± 16.4)**	**71.4 (± 15.7)**	**72.6 (± 14.9)**	**73.2 (± 16.4)**	**71.0 (± 18.6)**	**75.8 (± 13.3)**
**Symptoms Duration (months)**	**84.5 (± 108.6)**	**89.8 (± 114.9)**	**99.0 (± 134.8)**	**94.7 (± 123.4)**	**85.4 (± 108.0)**	**105.7 (± 142.1)**
**Age (years)**	**51.3 (± 14.4)**	**51.6 (± 14.1)**	**51.9 (± 14.7)**	**52.8 (± 14.3)**	**49.5 (± 17.0)**	**56.9 (± 8.8)**
Gender (female; male; other)	56; 12; 1	59; 8; 1	33; 5; 1	33; 3; 1	16; 3; 1	17; 0; 0
DHI	66.7 (± 16.6)	66.4 (± 16.0)	66.8 (± 16.1)	69.0 (± 15.0)	69.7 (± 15.8)	68.2 (± 14.6)
NPQ	36.7 (± 12.2)	34.5 (± 11.7)	35.5 (± 10.2)	35.2 (± 11.5)	36.3 (± 10.6)	33.8 (± 12.8)
HADS Anxiety	10.3 (± 4.2)	10.6 (± 4.1)	10.4 (± 4.0)	10.7 (± 3.7)	12.5 (± 3.4)	8.7 (± 3.1)
HADS Depression	8.8 (± 4.1)	8.9 (± 4.0)	8.8 (± 4.3)	9.6 (± 4.4)	10.9 (± 4.8)	8.2 (± 3.5)
Computer Use (Everyday; ~ 2 × a week; < 1 a week)	56; 7; 6	56; 10; 2	32; 5; 2	31; 5; 1	17; 3; 0	14; 2; 1

### Rehabilitation effects

**Figure 3:**
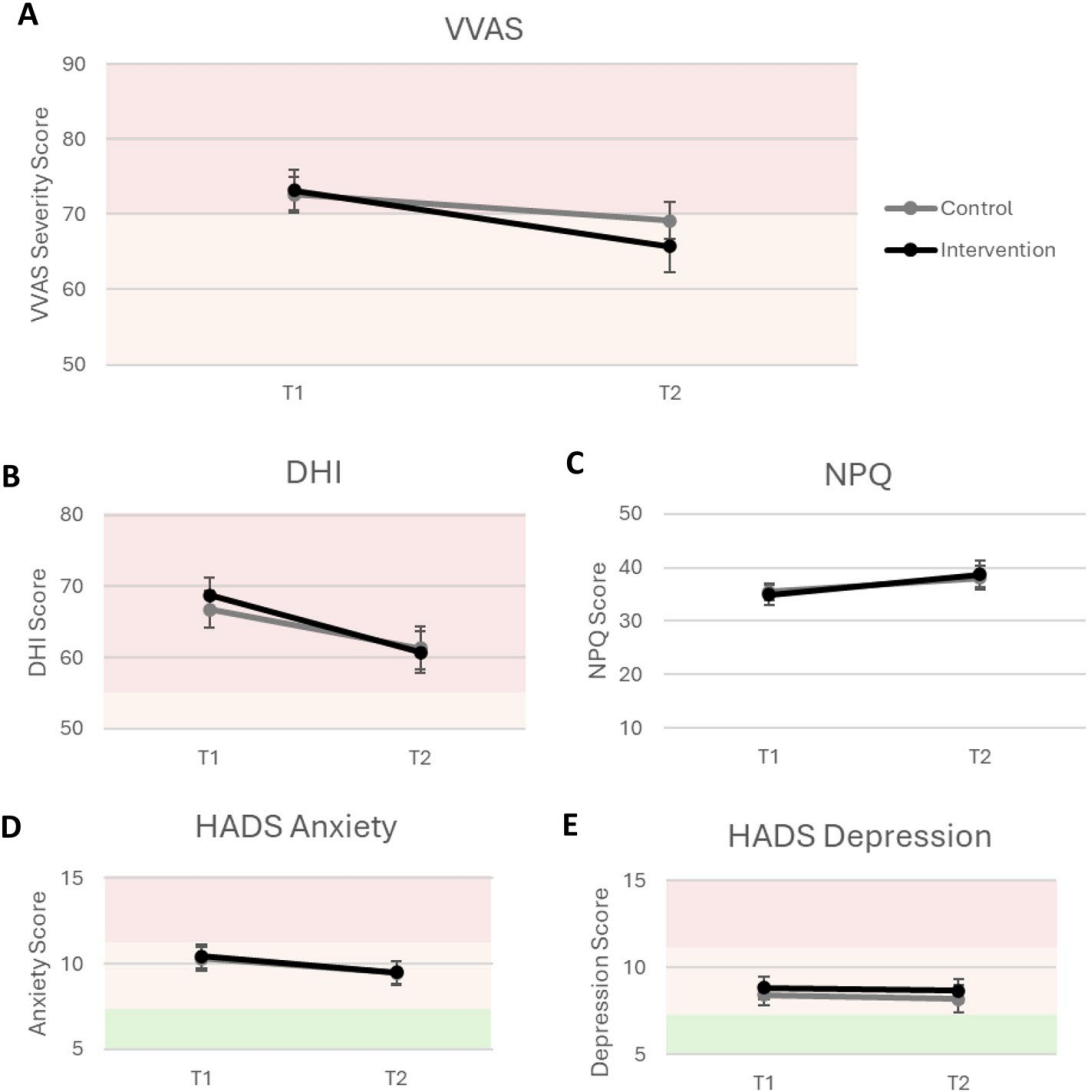
Intention to treat results for rehabilitation effects in primary outcome measure (VVAS, **A**) and secondary outcome measures: DHI (**B**), NPQ (**C**), HADS Anxiety (**D**), and HADS Depression (**E**) scores. Shaded areas indicate categories associated with each measure, where available (for VVAS and DHI, pink = severe, orange = moderate; for HADS, pink = clinically diagnosable, white = borderline, green = normal). Error bars are SEM.

**Figure 4:**
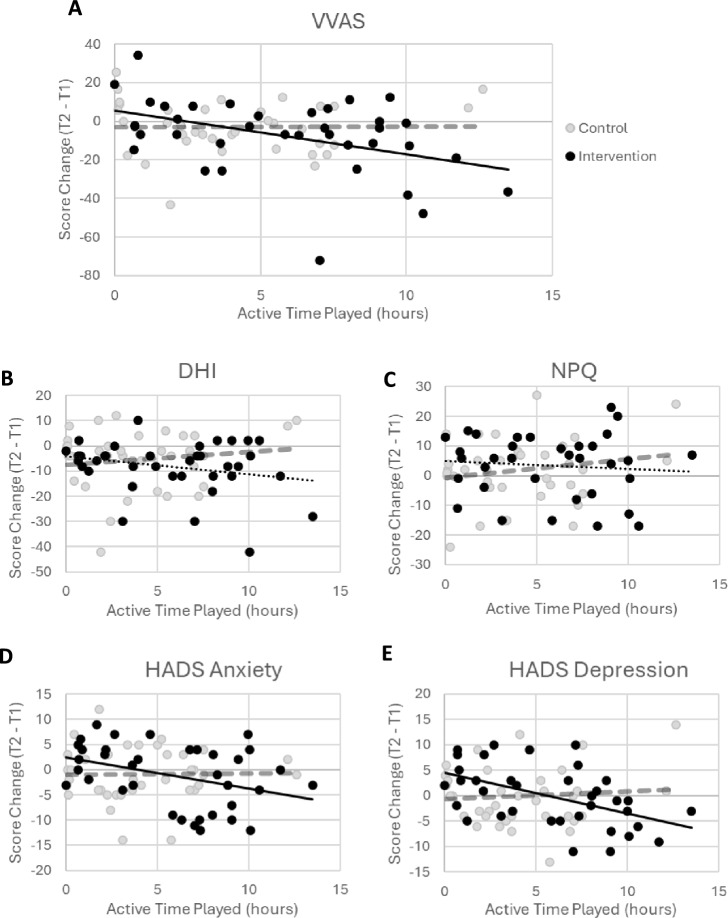
Correlations between active time played (intervention groups only) and changes in VVAS (**A**), DHI (**B**), NPQ (**C**), HADS Anxiety (**D**), and HADS Depression (**E**) between Time 1 and Time 2. Solid lines are significant correlations, dashed lines are non-significant. Black lines are intervention correlations and grey are control group correlations.

The primary outcome measure was VVAS severity scores. These reduced from a mean of 73.2 at time 1 to 65.8 at time 2 for the intervention group (Fig. 3A), with a smaller numerical reduction in the control group (72.6 to 69.1). More importantly, there was a clear correlation of this reduction with time spent playing the intervention game, but not for time spent playing the control condition (Fig. 4A, r(37) = − 0.43, 95% CI [− 0.66, − 0.12], see also Supplementary Fig. S1 for mean results for participants adhering to recommended playtime).

Our secondary dizziness and mental health measures are also plotted in Figs. 3 and 4. The dizziness handicap index (DHI) showed a reduction over time for both groups, largely independent of group or playtime (Figs. 3B and 4B). The Niigata PPPD Questionnaire (NPQ, Figs. 3C and 4C) increased slightly for both groups, independent of playtime. Anxiety and depression scores (Hospital Anxiety and Depression Scale, HADS) did not differ between groups in mean scores (Fig. 3D,E), but did reduce more for higher intervention playtime (Fig. 4D,E; *r*(38) = − 0.41, 95% CI [− 0.64, − 0.10],* r*(38) =  − 0.49, 95% CI [− 0.70, − 0.20], respectively), without correlating with control playtime (see also Supplementary Fig. S1 for mean results for participants adhering to recommended playtime).

As part of the time 2 surveys, participants provided qualitative responses to open-ended questions. Only responses from the intervention group (who played Balance-Land in full) are reported here. Several participants thought playing the game had helped (9), e.g. “*My symptoms have improved. Not completely gone, but I really do think the game has helped with the re-hab”;* “*I think I can tolerate more movement, more light, on screen and in life”;* “*I have noticed a significant improvement in my symptoms. I think a combined approach as listed above has definitely helped me’.* Some participants gave examples of improvements to their daily lives: *“Slightly less symptoms going through small stores; slightly improved ability to watch traffic at a busy intersection”*; *“Riding in a car has been better, I don’t get as sick as I once did”;* “*I seem to have a little more tolerance more movement of screens, although there are still some things, like flashing lights that [I] still can't tolerate.”* However, many participants (15) reported no major changes: *“I feel just as miserable as always, no changes to symptoms. I felt some minor improvement in the first few weeks of playing”*, or *“Yes after playing the game but overall no”*; *“Most of my symptoms have not changed”*, and others (5) were unsure: *“I generally feel less dizzy, but my dizziness always comes and goes in spells so it’s hard to know what it's down to”*; *“Not sure if it's just coincidence but since playing the game I have had far less very bad days in general.”* Some participants mentioned anxiety reduction or improved understanding “*I'm less scared of PPPD now”;* “*I feel like I'm more aware of what the triggers are from playing the game.”*

### System usability and enjoyment

**Figure 5:**
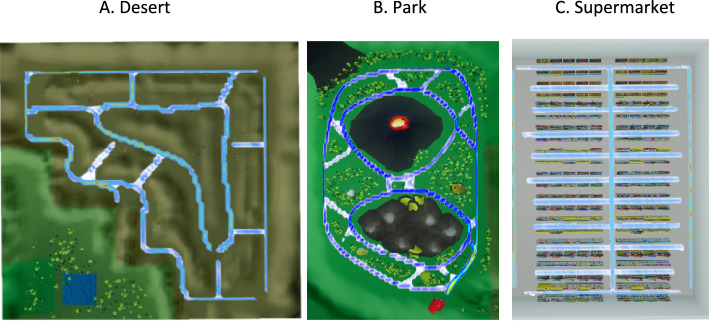
Heatmap of paths taken by participants, overlayed on the three zones of Balance-Land. Participants accessed all areas and used all available pathways. The supermarket had denser inputs due to participants frequently inputting commands to turn down aisles, compared to the looping paths of the other environments. Participants tended not to input commands on longer curved paths.

We used the System Usability Scale (SUS^24^) to measure usability. The mean score for the intervention group was 80 (12.8 SD [45, 100]), which is equivalent to the 80th percentile of usability (categorised as ‘highly useable’^25^). There was no difference between this group and the Control group who only played the word games without having to navigate the virtual zones (*F*(2,74) = 0.73). There were no correlations of SUS with the outcome measures reported above, suggesting that differences in usability do not account for the rehabilitation effects reported. To assess whether all areas of the game were accessible to participants, we compared the locations of player inputs to a map of available locations across the zones (Fig. 5). Player inputs were recorded in all usable locations.

Participants were asked at time 1 (before accessing Balance-Land) about their digital experience, and of the participants that completed the study, 64 reported using computers nearly every day, while 13 reported using computers about twice a week or less. Only 6 of these infrequent users were in the intervention group and they were as likely to play for the recommended playtime (3) as not (3), indicating no evidence that low digital experience explains low playtime. In exit interview responses, participants reported learning the game as straightforward or easy (15/15), and most (9/15) reported that they had learnt to use the controls within the first session.

Participants were asked to rate their enjoyment from 0 (none) to 10 (high). The mean ratings were 5.9 for control and 7.2 for intervention (7.8 for those playing the recommended time, 6.6 for those with low playtime). There were no significant correlations of enjoyment score with outcome measures for the Intervention Group. In response to the open-ended questions, some participants (6) spontaneously mentioned finding the game enjoyable: *“The games were great and definitely got easier over time”*. However, some participants expressed frustration (5) with certain aspects of gameplay and many (10) reported difficulty fitting in or sticking to the recommended schedule *“Hard to do as life always interfered!”*. Others reported that they needed reminders *“5–10 min was a short amount of time to dedicate out of my day, which made it easy to integrate into my routine. However, it was easy to forget to play the game, especially if my daily routine changed.”*

The participants’ final open-ended question was whether they would play Balance-Land more if there was evidence it reduced symptoms, and how often they would play. Some participants said that the twice-daily playtime we recommended in the trial was enough (12), with reasons relating to daily life *“Absolutely. Twice daily unless unusual circumstances prevent me from doing so”,* and symptom load *“I do not think that I could tolerate playing more than I did and still be able to do other things throughout my day.”* However, the majority (20) responded that they would play more: *“3–4 Times a Day”* and “*yes. I would play at least an hour a day”*, *“Sure, as often as [I] remember to” “Yes I would play 24/7 if I have [to]”* and *“Yes, as often as it took”*. Only 2 participants responded that they would not play the game, and this was because it had not triggered symptoms for them.

### Daily dizziness

**Figure 6:**
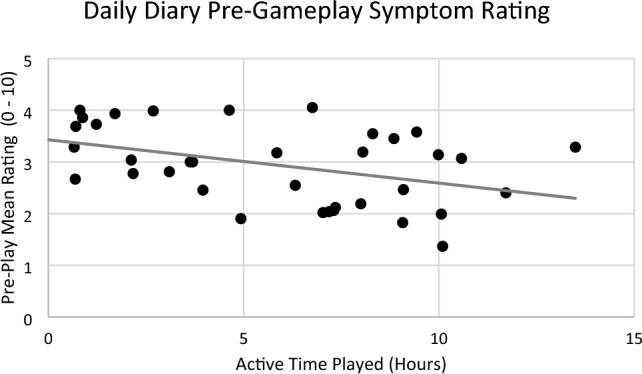
The mean of participants’ daily symptom rating before-gameplay (how severe symptoms were before playing) correlated with their active time played in hours (plotted for intervention groups).

To determine whether engagement with the game was associated with symptom severity, initial VVAS score and daily symptom ratings were correlated with active playtime for the intervention group. There was no correlation of playtime with Time 1 VVAS symptoms (*r*(37) = 0.26), but there was a correlation with the daily diary symptom ratings (Fig. 6, *r*(36) = − 0.41), such that people with a lower daily symptom rating before playing Balance-Land tended to play for longer or more often. There was no correlation between playtime and daily post-play symptom rating (*r*(35) = − 0.24). In the daily diaries we also asked if participants had done other vestibular rehabilitation; however, this data predicted neither game playtime nor evoked symptoms.

## Discussion

Debilitating visually-induced dizziness, such as occurs in PPPD and other conditions, is very difficult to treat. In this online feasibility study we aimed to assess the potential usefulness of embedding visual desensitisation within a game context to allow graded exposure to visual motion in virtual environments and allow everyday usage at home. If useful, this approach could become a rehabilitation option as part of a wider treatment package.

### Rehabilitation effectiveness

The reduction in visual vertigo symptoms (VVAS) in participants who chose to play Balance-Land for the recommended time converge with prior findings^7^ that viewing visual flow patterns reduced symptoms of visually-induced dizziness. Interestingly, daily diary symptom levels before and after gameplay were not significantly different between the intervention (visual flow) and control (no visual flow), meaning that although control condition puzzle games triggered some symptoms, merely triggering symptoms with non-motion stimuli may not be sufficient for rehabilitation^4^.

We also found that anxiety decreases correlated with playtime for the intervention group, but not the control. This reduction in anxiety scores was at a clinically meaningful level for many participants engaging with the game recommendations. Anxiety is known to be a strong precipitating and maintaining factor in PPPD^1,4,26^ and a correlate of visually-induced dizziness and sensitivity to visual stimuli across all conditions where it arises, as well as in the general population^27^. Therefore, supporting improvements in anxiety may be as important for rehabilitation and quality of life as targeting dizziness itself. Recently, other research has highlighted the beneficial effect targeting anxiety can have for PPPD recovery^8^, and many clinicians who provided feedback on game development reported prioritising anxiety treatment ahead of vestibular exercises for dizzy patients. The qualitative data indicate that anxiety reduction may be a consequence both of becoming more self-aware of triggers, and of being exposed to triggering environments in a safe and controlled manner (with easy escape).

We also found some differential reduction in depression scores, which is often correlated with anxiety. We did not find differential rehabilitation effects in the DHI or NPQ scores. The reasons for this remain unclear and could not be explained by separating the NPQ into subscales (visual vs postural^28^) or incomplete answers for our diverse participant group^29^. Of note, the VVAS score change correlated with the DHI score change (*r*(37) = 0.55, 95% CI [0.27, 0.74]) and the NPQ score change (*r*(34) = 0.60, 95% CI [0.31, 0.77]), as they would broadly be expected to, providing no evidence that one or more questionnaires were being filled out incorrectly.

Taken together, we have preliminary evidence, albeit with a small sample size and only in people self-selected for playtime, that visual desensitisation within a game environment may be effective in lowering visually-induced dizziness and anxiety. The mechanism is most likely to be the same as the visual optokinetic paradigms that inspired the creation of Balance-Land^4,7,30–32^. Future research is needed to confirm these effects in a larger population and to see if they are maintained over a longer time frame.

### Attrition, adherence and self-selection

Attrition and adherence to recommended playtime were clear challenges in the feasibility study and need to be further addressed to enable future research or clinical use of Balance-land. Overall, 45% of participants who completed Time 1 assessments withdrew before completing Time 2. This is not unusual for unsupervised rehabilitation (e.g. Pavlou et al.^7^ report 55% dropout in their unsupervised group). For those who did complete Time 2, ten participants mentioned difficulties fitting the sessions into their daily schedules and less than half of participants played the recommended amount (at least 10 min a day on average). It is worth noting that vestibular rehabilitation is normally recommended for 10 min a day as a minimum (unless severe acute symptoms prevent this), and without such time commitment rehabilitation progress would not be expected.

Importantly, we warned participants at the beginning of the study that there was currently no evidence that Balance-Land could improve symptoms, and this is a key factor that likely affected motivation. Of the intervention participants responding to the exit survey, 34 said they would be happy to play Balance-Land at the recommended dosage if evidence suggested it could improve symptoms, and 22 of these said they would play for longer than the 5–10 min twice daily we recommended in the study. Therefore, it appears that a key ‘*chicken and egg*’ difficulty for research engagement is the lack of such evidence beforehand.

For participants who withdrew, the reason for withdrawal was not known in many cases and could have created bias. Of the reasons reported, common reasons were co-occurring health issues (6), technical difficulties (3), and time commitment (2). Two participants withdrew because they attempted to play levels within the game that were too intense and triggered too many symptoms. However, overall withdrawal was not related to VVAS severity at Time 1 or digital experience. Of the unknown reasons, it is possible that participants who did not think that Balance-Land was reducing their symptoms were more likely to withdraw, potentially creating a self-selection bias in the findings. This kind of attrition is common in both home settings and hospital-based rehabilitation therapies^31^. One advantage here was that many participants reported not knowing which group they were in (they did not unblind themselves based on what kinds of gameplay they saw), making it less likely that unknown attrition reasons were markedly different across groups.

However, a second kind of self-selection was introduced by whether participants played for the recommended time or not. To meaningfully assess rehabilitation promise, we assessed correlations with playtime (and in supplementary information, we plot results for adhering participants only, breaking the randomisation of participants). Fortunately, those who played or did not play for the recommended time did not show major differences in VVAS severity, duration of symptoms, or age, in any direction likely to account for the group differences we found (Table 1). However, there may be other differences between participants that influenced, or correlated with, their chosen playtime. For example, perceiving that their dizziness was improving may have been a motivation to keep playing, rather than (or as well as) a consequence of playing. Another difference is in the proportions of reported diagnoses (see Supplementary Information, albeit with the caveat that diagnoses are not always correct, as discussed above). Numerically more participants with low playtime reported a PPPD diagnosis, which is known to be difficult to rehabilitate. Although PPPD is one of the key conditions targeted by the game, the complexities of PPPD and other comorbid conditions will impact rehabilitation success and/or may make engaging with the game more difficult.

The daily dizziness ratings may partially explain differences in adherence, where lower pre-play symptoms correlated with higher playtime. This may be interpreted as indicating that higher daily symptoms are a barrier to engaging with symptom-provoking rehabilitation (though note that daily symptom ratings may also partly reflect the improvements over time for those playing the game more). One of the key aims of Balance-land was graded stimulation to allow an entry point to rehabilitation even for those with severe symptoms. A graded and slow build-up may need to be better explained and planned for participants in future. We did not block participants from quickly engaging in the more complex zones or using faster speeds and some participants chose levels that they could not tolerate in the very first play sessions (despite advice not to).

### Digital accessibility and enjoyment

The System Usability Scale (SUS) scores and the exit interviews indicated Balance-Land was accessible to a range of users, across a range of ages and digital experience. We identified no barrier for those with lower digital experience. However, participants who struggled with accessibility may have withdrawn from the study, although only one person gave this as a reason for their withdrawal. Participants were recruited online and thus self-selected for some degree of computer use. Interestingly, participants who found Balance-Land more useable also reported experiencing more symptoms after playing it. One explanation for this might be that better understanding of how to play the game helped people to more effectively expose themselves to visual flow and trigger symptoms. Reassuringly, enjoyment was rated moderately highly, although a game aimed primarily at rehabilitation is not ever likely to be as enjoyable as commercial games aimed primarily at enjoyment. The puzzle game play was chosen to engage older demographics^33,34^ and we interviewed participants directly during development^12^. We also know that immersion aids enjoyment and motivation^35^, which was one of the reasons for aiming to mimic real-life environments.

### Balance-land in practice

The aim of Balance-Land is not to replace other kinds of rehabilitation therapy. We hope that Balance-land can become part of a multi-faceted treatment approach for patients experiencing visually-induced dizziness. It utilises already-evidenced principles of optic flow desensitisation and we have found no indication of detrimental effects, other than the symptoms expected to be evoked by rehabilitation.

A key advantage of Balance-Land is that rehabilitation intensity can be increased gradually. Recommended playtime and intensity of visual exposure will need to be calibrated for different patients and potentially built up in a rehabilitation schedule over several weeks, exactly as current visual desensitisation therapy is scheduled. We will therefore put tighter controls in place to limit access to the higher levels of intensity until players have built up experience in the game. We recommended playtime of 5–10 min twice a day in the trial based on discussions with clinicians and our patient advisory group about likely feasibility and symptom evocation, but it is likely that higher dosage would be desirable if tolerated. For example, Pavlou et al.^7^ used up to 45 min a day for optokinetic desensitisation.

Importantly, Balance-Land is a web-based application requiring no specialist equipment. Ideally, Balance-land should be played on the largest screen available to patients to maximise visual field, but some participants in our study used tablets (presumably with shorter viewing distance, but this was not measured). Balance-Land can be adapted to work on phones, however the necessary field of view for visual desensitisation is not known.

## Conclusion

The goals of this feasibility trial were to assess Balance-Land’s: usage in a varied cohort with visually-induced dizziness; rehabilitation potential; system usability and enjoyment; relationship with daily symptoms. Around half of participants completed 6 weeks of playing Balance-Land, and about half of those played for at least an average of 10 min a day. For the latter group, there appeared to be rehabilitation effects in reduced visually-induced dizziness, anxiety and depression, though other explanations are possible given they self-selected for gameplay time. System usability was high and some participants with relatively low digital experience engaged successfully with the game. Moderate enjoyability was reported, but like all vestibular rehabilitation, Balance-Land evokes symptoms and those with higher daily symptoms tended to play less, suggesting that managing symptom load through even more graded exposure is critical for engagement. Further research is required in a clinical setting.

## Methods

### Balance-Land—description and development

Players move through virtual environments collecting letters for word games or collecting items from a shopping list. Different zones (Fig. 1) provide different intensities of optokinetic stimulation: the Desert zone is low contrast and spatial frequency, with a limited colour palette and few objects; the Park zone steps up these characteristics, with tree trunks and bushes; the Supermarket zone is high contrast and spatial frequency, brightly coloured, with many objects (chosen to simulate a common situation where patients have difficulties^13,14^. Within each zone, players can change the movement speed and steadiness, choose to enter more visually complex areas, and choose puzzle settings that provide more or fewer breaks from visual stimulation.

Word games and shopping lists were selected as accessible to a wide range of users without requiring experience with computer game controls^33^. Puzzle games also crucially allow for the gameplay difficulty (i.e. puzzles) to be decoupled from the difficulty of the rehabilitation (e.g. speed and complexity of optokinetic stimulation). A web-based platform was used to ensure access from different types of devices, as well as enabling gameplay information to be recorded.

Balance-Land was developed over three rounds of iterative feedback, via questionnaires and interviews, with patients and clinicians^12^. In total, 21 people with PPPD and visually-induced dizziness symptoms and 6 clinicians helped to design and optimise all aspects of the game. This development process established the need for Balance-Land, helped to ensure user-accessibility and enjoyment, and provided insight into how to titrate rehabilitation intensity.

### Design

The study was designed with two parallel groups, pseudo-randomised to match groups on key characteristics, with an assessment before (Time 1) and after (Time 2) 6 weeks of access to Balance-Land or a control version of the game without optic flow. Participants were additionally invited to a structured qualitative interview after Time 2. All qualitative data was analysed with content analysis^36^.

### Setting and participants

All methods were carried out in accordance with relevant guidelines and regulations. Experimental protocols were approved by the Ethical Committee of the School of Psychology, Cardiff University. Written informed consent was obtained from all participants.

Participants took part online and were able to play the game at home on their own laptop, computer, or tablet. Adults (aged 18 or over) were recruited online through VEDA (https://vestibular.org), the Meniere’s Society (https://www.menieres.org.uk), and social media. Volunteers were initially screened with the Visual Vertigo Analogue scale (VVAS) in which nine environments and triggers (commonly associated with visually-induced dizziness) are rated from 0–10 for the degree they evoke dizziness^13^. Volunteers were invited to take part if their severity score exceeded 40 (moderate^37^; see Fig. 2 for a recruitment pipeline). Participants had to be able to read and understand English but were not excluded on other criteria and any person with an internet connection was eligible to join.

We based recruitment on VVAS severity scores rather than current diagnoses for three reasons: visually-induced dizziness occurs across more than one condition; visual desensitisation is aimed at the experience of visually-induced dizziness, rather than being expected to treat all aspects of a condition, such as PPPD; diagnosis for dizziness-related conditions is notoriously difficult and often incorrect (high levels of misdiagnosis have been reported across Europe, USA, and China^15,16,38,39^, concurring with our clinical experience).

### Randomisation

Participants were pseudo-randomised in batches in order to match the intervention and control groups on three factors: VVAS severity score, age, and duration of symptoms, prioritised in that order (see Table 1 for participant information), and in order that volunteers were not kept waiting more than a week to join the study. In other words, the first pair were randomly allocated to different groups, and then for all possible permutations of allocation for the rest of the batch, the difference in mean VVAS score between the groups was calculated and the permutation selected that minimised this difference (using Excel for Microsoft 365 v2406). If more than one permutation offered acceptable matching (1 point difference or less), then difference in mean age was minimised. If more than one permutation kept mean age difference below 1 year, then difference in mean illness duration was also minimised. The researcher was blind to all other participant information at allocation. After allocation, all interaction with the data was via participant ID codes that did not reveal the group. This also allowed the researcher to provide technical support and to perform exit interviews without knowing the group (although some participants revealed their group through their comments).

### Procedure

Both groups were recommended to play for (no more than) 5–10 min twice daily (symptoms allowing) for 6 weeks. Participants were asked not to make any adjustments to any current treatment plan or any other activities relevant to their symptoms, but to simply play the game in addition. Participants were told that Balance-Land might trigger symptoms, and to pause, take a break, or stop playing, depending on the severity of the symptoms. The instructions were “The goal is to evoke **MILD** symptoms. If you are experiencing more than this, please lower the speed, go to a simpler environment, or take a break.”

### Intervention group

Participants in the intervention group played Balance-Land. During each game session they could freely choose between playing three virtual environments: Desert, Park, and Supermarket (see Fig. 1). There were three possible game modes: Find a word; Make a word; and Shop (only available in the Supermarket). Participants were not restricted in what they could access, but they were advised to keep symptom triggering at a comfortable level rather than over-stimulate (going to the Supermarket too soon, for example). Participants were given a series of short (< 2 min) video tutorials that covered how to play the game and the options available. These tutorials remained available via a link in-game.

### Control group

Participants in the control condition played a modified version of Balance-Land, with no virtual environments to move within, thus eliminating the optic flow aspects of gameplay. They could play two game modes: Find a word; and Make a word, but letters were provided and did not need to be found within the virtual environments. Participants were given a series of short (< 2 min) video tutorials that covered how to play the games (which remained available via a link in-game).

### Feasibility outcomes and planned analyses

#### Dropout and adherence

We offered each participant who stopped playing the game during the trial (no gameplay for a week), or who never played a single session, the opportunity to provide a reason. We measured the active time played by each participant, defined using any input 30 s or less from another input (to remove instances where participants left the game running whilst not being used, for example if they were taking a break or in order to carry on from the same stage the next day).

#### Rehabilitation effect

We used VVAS score (outlined above) as the primary outcome measure, assessing the change between time 1 and time 2 and dependency on total game playtime (assessed with ANOVA and correlation). Secondary outcome measures were also included: Dizziness Handicap Inventory (DHI^40^), the Niigata PPPD Questionnaire (NPQ^14^), and the Hospital Anxiety and Depression Scale (HADS^41^). All surveys were delivered in Qualtrics^42^. At Time 2 participants were able to provide open-ended answers about their experiences, as well as sign up for a post-trial interview.

#### Digital accessibility and usability

To assess usability participants completed the System Usability Scale (SUS^24^) at Time 2 only. As secondary outcomes, we used gameplay data to assess whether participants accessed all virtual environments and controls, and asked how quickly they learnt the controls. We particularly focussed on participants with lower digital experience (Participants reported level of digital experience at time 1 only: every day, ~ 2 × a week, < 1 a week).

#### Enjoyment

Participants rated their enjoyment of the game out of 10 at time 2 and provided qualitative feedback (if they participated in the interview).

#### Daily symptom diary

We used a simple brief rating scale to assess daily symptoms so that we could assess whether this predicted how much participants engaged with the game. For each session, participants were asked: “Before playing Balance-Land today, how severe are/were your symptoms?”; and “After playing Balance-Land today, how severe are/were your symptoms?”. Participants selected one of six faces that progressively changed from smiling to frowning and crying. We also asked if they had performed other vestibular rehabilitation (yes/no).

#### Statistical methods

We used descriptive statistics, 95% CI, correlation and ANOVA (see Supplementary Fig. S1 for ANOVA results), using Jamovi (version 2.3.28)^43^ and SPSS (version 27)^44^. We do not give p values in order not to overemphasise significance in a feasibility study. Figures were plotted using Screencaps, Excel and Powerpoint for Microsoft 365 v2406, Matlab R2023A (https://uk.mathworks.com/products/new_products/release2023a.html).

### Supplementary Information

Supplementary Information.

## Data Availability

The data are freely available here: https://osf.io/5y279/.

## References

[1] Staab, J. P. *et al.* Diagnostic criteria for persistent postural-perceptual dizziness (PPPD): Consensus document of the committee for the Classification of Vestibular Disorders of the Barany Society. *J. Vestib. Res.* **27**, 191–208. 10.3233/VES-170622 (2017). 29036855 10.3233/VES-170622 PMC9249299

[2] Powell, G., Derry-Sumner, H., Rajenderkumar, D., Rushton, S. K. & Sumner, P. Persistent postural perceptual dizziness is on a spectrum in the general population. *Neurology* **94**, e1929–e1938 (2020). 32300064 10.1212/WNL.0000000000009373 PMC7274923

[3] Bronstein, A. M. The visual vertigo syndrome. *Acta Oto-Laryngol. Suppl.* **520 Pt 1**, 45–48 (1995). 10.3109/00016489509125186 8749077

[4] Popkirov, S., Stone, J. & Holle-Lee, D. Treatment of persistent postural-perceptual dizziness (PPPD) and related disorders. *Curr. Treat. Options Neurol.* **20**, 50. 10.1007/s11940-018-0535-0 (2018). 30315375 10.1007/s11940-018-0535-0

[5] Pavlou, M., Lingeswaran, A., Davies, R. A., Gresty, M. A. & Bronstein, A. M. Simulator based rehabilitation in refractory dizziness. *J. Neurol.* **251**, 983–995 (2004). 15316804 10.1007/s00415-004-0476-2

[6] Yardley, L., Masson, E., Verschuur, C., Haacke, N. & Luxon, L. Symptoms, anxiety and handicap in dizzy patients: Development of the vertigo symptom scale. *J. Psychosom. Res.* **36**, 731–741 (1992). 1432863 10.1016/0022-3999(92)90131-K

[7] Pavlou, M., Bronstein, A. M. & Davies, R. A. Randomized trial of supervised versus unsupervised optokinetic exercise in persons with peripheral vestibular disorders. *Neurorehabil. Neural Repair* **27**, 208–218. 10.1177/1545968312461715 (2013). 23077146 10.1177/1545968312461715

[8] Herdman, D. *et al.* The INVEST trial: A randomised feasibility trial of psychologically informed vestibular rehabilitation versus current gold standard physiotherapy for people with Persistent Postural Perceptual Dizziness. *J. Neurol.* **269**, 4753–4763 (2022). 35397754 10.1007/s00415-022-11107-w PMC8994825

[9] Gamble, R. *et al.* Using interpretative phenomenological analysis to probe the lived experiences of persistent postural-perceptual dizziness (PPPD). *J. Vestib. Res.* **33**, 89–103 (2023). 36710692 10.3233/VES-220059 PMC10041438

[10] Whitney, S. L., Sparto, P. J. & Furman, J. M. *Seminars in Neurology* 165–172 (Thieme Medical Publishers, 2022).

[11] Sardi, L., Idri, A. & Fernández-Alemán, J. L. A systematic review of gamification in e-Health. *J. Biomed. Inform.* **71**, 31–48 (2017). 28536062 10.1016/j.jbi.2017.05.011

[12] Goodwin, N. *et al.* Balance-Land: A gamified rehabilitation program for people with Persistent Perceptual Postural Dizziness (PPPD) and visual vertigo [Online]. *PsyArXiv Center for Open Science*10.31234/osf.io/9gb73 (2023). 10.31234/osf.io/9gb73

[13] Dannenbaum, E., Chilingaryan, G. & Fung, J. Visual vertigo analogue scale: An assessment questionnaire for visual vertigo. *J. Vestib. Res. Equilib. Orient.* **21**, 153–159 (2011). 10.3233/VES-2011-0412 21558640

[14] Yagi, C. *et al.* A validated questionnaire to assess the severity of persistent postural-perceptual dizziness (PPPD): The Niigata PPPD Questionnaire (NPQ). *Otol. Neurotol.* **40**, e747–e752. 10.1097/MAO.0000000000002325 (2019). 31219964 10.1097/MAO.0000000000002325 PMC6641087

[15] To-Alemanji, J., Ryan, C. & Schubert, M. C. Experiences engaging healthcare when dizzy. *Otol. Neurotol.* **37**, 1122–1127 (2016). 27525623 10.1097/MAO.0000000000001145

[16] Thomson, P. Ménière’s: Why its diagnosis calls for more careful evaluation. *Br. J. Gen. Pract.* **67**, 569–570 (2017). 29192115 10.3399/bjgp17X693821 PMC5697545

[17] Axer, H. *et al.* Multimodal treatment of persistent postural–perceptual dizziness. *Brain Behav.* **10**, e01864 (2020). 32989916 10.1002/brb3.1864 PMC7749543

[18] Zhang, L., Jiang, W., Tang, L., Liu, H. & Li, F. Older patients with persistent postural-perceptual dizziness exhibit fewer emotional disorders and lower vertigo scores. *Sci. Rep.* **12**, 11908 (2022). 35831350 10.1038/s41598-022-15987-w PMC9279357

[19] Chari, D. A., Liu, Y.-H., Chung, J. J. & Rauch, S. D. Subjective cognitive symptoms and dizziness handicap inventory (DHI) performance in patients with vestibular migraine and Menière’s disease. *Otol. Neurotol.* **42**, 883–889 (2021). 33606474 10.1097/MAO.0000000000003081

[20] Kim, T. S., Lee, W. H. & Heo, Y. Prevalence and contributing factors of anxiety and depression in patients with vestibular migraine. *Ear Nose Throat J.*10.1177/01455613231181219 (2023). 10.1177/01455613231181219 37329273

[21] Bruderer, S. G., Bodmer, D., Stohler, N. A., Jick, S. S. & Meier, C. R. Population-based study on the epidemiology of Ménière’s disease. *Audiol. Neurotol.* **22**, 74–82 (2017). 10.1159/000475875 28723686

[22] Kirby, S. E. & Yardley, L. Cognitions associated with anxiety in Ménière’s disease. *J. Psychosom. Res.* **66**, 111–118 (2009). 19154853 10.1016/j.jpsychores.2008.05.027

[23] Söderman, A.-C.H., Bagger-Sjöbäck, D., Bergenius, J. & Langius, A. Factors influencing quality of life in patients with Ménière’s disease, identified by a multidimensional approach. *Otol. Neurotol.* **23**, 941–948 (2002). 12438860 10.1097/00129492-200211000-00022

[24] Brooke, J. SUS-A quick and dirty usability scale. *Usability Evaluation in Industry* **189** (1996).

[25] Lewis, J. R. & Sauro, J. Item benchmarks for the system usability scale. *J. Usability Studies* **13**, 38–46 (2018).

[26] Staab, J. P., Rohe, D. E., Eggers, S. D. & Shepard, N. T. Anxious, introverted personality traits in patients with chronic subjective dizziness. *J. Psychosom. Res.* **76**, 80–83. 10.1016/j.jpsychores.2013.11.008 (2014). 24360146 10.1016/j.jpsychores.2013.11.008

[27] Powell, G. *et al.* Visually-induced dizziness is associated with sensitivity and avoidance across all senses. *J. Neurol.* **267**, 2260–2271 (2020). 32306170 10.1007/s00415-020-09817-0 PMC7359147

[28] Yagi, C. *et al.* Subtypes of persistent postural-perceptual dizziness. *Front. Neurol.* **12**, 652366 (2021). 33935950 10.3389/fneur.2021.652366 PMC8085253

[29] Castillejos-Carrasco-Muñoz, R. *et al.* Validity and reliability of the Niigata PPPD Questionnaire in a Western population. *Eur. Arch. Oto-Rhino-Laryngol.* **280**, 5267–5276 (2023). 10.1007/s00405-023-08038-1 PMC10620260 37266755

[30] Mandour, A.E.-S., El-Gharib, A. M., Emara, A. A. & Elmahallawy, T. H. Virtual reality versus optokinetic stimulation in visual vertigo rehabilitation. *Eur. Arch. Oto-Rhino-Laryngol.* **279**, 1609–1614 (2022). 10.1007/s00405-021-07091-y 34611745

[31] Teh, C.S.-L. *et al.* Home-based vestibular rehabilitation: A feasible and effective therapy for persistent postural perceptual dizziness (a pilot study). *Ann. Otol. Rhinol. Laryngol.* **132**, 566–577 (2023). 35794811 10.1177/00034894221111408

[32] Nada, E. H., Ibraheem, O. A. & Hassaan, M. R. Vestibular rehabilitation therapy outcomes in patients with persistent postural-perceptual dizziness. *Ann. Otol. Rhinol. Laryngol.* **128**, 323–329 (2019). 30607985 10.1177/0003489418823017

[33] Salmon, J. P. *et al.* A survey of video game preferences in adults: Building better games for older adults. *Entertain. Comput.* **21**, 45–64 (2017). 10.1016/j.entcom.2017.04.006

[34] Chesham, A., Wyss, P., Müri, R. M., Mosimann, U. P. & Nef, T. What older people like to play: Genre preferences and acceptance of casual games. *JMIR Serious Games* **5**, e8 (2017). 28420601 10.2196/games.7025 PMC5413800

[35] Weibel, D. & Wissmath, B. Immersion in computer games: The role of spatial presence and flow. *Int. J. Comput. Games Technol.* **2011**, 6–6 (2011). 10.1155/2011/282345

[36] Elo, S. & Kyngäs, H. The qualitative content analysis process. *J. Adv. Nurs.* **62**, 107–115 (2008). 18352969 10.1111/j.1365-2648.2007.04569.x

[37] Frank, A. J., Hoppes, C. W., Dunlap, P. M., Costa, C. M. & Whitney, S. L. Categorizing individuals based on the severity of visual vertigo analogue scale symptoms. *J. Vestib. Res.* **32**, 433–441 (2022). 35466914 10.3233/VES-210131

[38] Van Leeuwen, R. B. & Van Der Zaag-loonen, H. Referrals to a specialised dizziness clinic often result in revised diagnoses and new therapeutic advice. *Eur. Neurol.* **73**, 20–22 (2015). 25376792 10.1159/000366415

[39] Jin, Z., Zhuang, J., Zhao, Z., Chen, Y. & Li, Y. Analysis of misdiagnosed cases with benign paroxysmal positional vertigo. *Zhonghua yi xue za zhi* **92**, 1346–1348 (2012). 22883126

[40] Jacobson, G. P. & Newman, C. W. The development of the dizziness handicap inventory. *Arch. Otolaryngol. Head Neck Surg.* **116**, 424–427 (1990). 2317323 10.1001/archotol.1990.01870040046011

[41] Zigmond, A. S. & Snaith, R. P. The hospital anxiety and depression scale. *Acta Psychiatr. Scand.* **67**, 361–370 (1983). 6880820 10.1111/j.1600-0447.1983.tb09716.x

[42] Qualtrics v. March 2023 (Qualtrics, https://www.qualtrics.com, 2005).

[43] The jamovi project v. 2.3.28 (https://www.jamovi.org, 2024).

[44] IBM SPSS Statistics for Windows v. 26.0 (IBM Corp, 2020).

