# Supplementary material for: Feasibility of gamified visual desensitisation for visually-induced dizziness
Source: Sci Rep. 2024 Aug 1;14:17864. doi: 10.1038/s41598-024-67745-9 (PMC11294564; doi:10.1038/s41598-024-67745-9)

Feasibility of gamified visual desensitisation for visually-induced dizziness

Supplementary information

Table S1. Balance-related diagnoses reported by participants that completed time 2 (and in brackets for participants at time 1). (*) indicates PPPD diagnoses included prior diagnosis labels, e.g. visual vertigo. We asked participants to tick all that applied for a list of conditions, or to supply other 'balance-related' diagnoses. Participants were recruited globally online from the following countries: USA (60), UK (51), Canada (12), Australia (2), Norway (2), Italy (2), Brazil (1), Finland (1), Germany (1), India (1), New Zealand (1), and South Africa (1). At Time 2, this was reduced to: USA (25), UK (29), Canada (6), Australia (1), Norway (2), Italy (1), Finland (1), and South Africa (1).

Diagnosis	Control<br>(n=39) (t1 n = 70)	Intervention<br>(n=37) (t1 n = 68)	Recommended Playtime<br>(n=17)	Low Playtime<br>(n=20)
**PPPD***	16 (29)	21 (37)	7	14
**Vestibular Migraine**	17 (35)	14 (26)	6	8
**Meniere's Disease**	7 (11)	3 (7)	1	2
**Vestibular Neuronitis**	3 (5)	5 (6)	4	1
**BPPV**	2 (4)	5 (9)	4	1
**Bilateral Vestibular Loss**	4 (7)	0 (4)	0	0
**Labyrinthitis**	1 (2)	0 (1)	0	0
**Vertigo from head trauma**	3 (5)	1 (2)	0	1
**Dizziness or vertigo due to stroke**	1 (2)	0 (0)	0	0
**Vestibular Schwannoma**	0 (1)	1 (2)	1	0
**Mal de Debarqument (MDDS)**	0 (1)	0 (2)	0	0
**Other**	7 (15)	2 (9)	0	2
	1. Anxiety<br>2. Cervical vertigo<br>3. Endolymphatic hydrops<br>4. Vestibular tone imbalance, Peripheral Vestibular Disfunction<br>5. Binocular vision dysfunction, cervical spine dysfunction<br>6. Covid<br>7. Vestibular dysfunction	1. Autoimmune inner ear disease<br>2. Nerve damage due to lyme		1. Autoimmune inner ear disease<br>2. Nerve damage due to lyme

Table S2. Measured characteristics at screening stage for participants invited to enrol (meeting criteria for VVAS score and age) who joined or did not join the study.

	Participants who enrolled N = 138	Participants who declined N = 233
VVAS Severity	70.8	65.4
Age (years)	51.5	54.1
Gender % (female; male; other)	84;15;1	88; 11; 1
%PPPD	48	8
% Vestibular migraine	44	44
% Meniere's Disease	13	21
% Vestibular Neuronitis	8	16
% BPPV	9	15

Table S3. Comparison between current study participants with PPPD, Vestibular Migraine and Meniere's Disease and published cohorts (selected for large N where available; other published cohorts tend to fall within the SD ranges below when N is sufficient).

	Study cohort at T1			Literature		
	PPPD	Vestibluar migraine	Meniere's Disease	PPPD[1]	Vestibluar migraine	Meniere's Disease
Age	50 ± 16	51 ± 13	51 ± 17	50 ± 14 [1]	46 ± 15[3]	55 ± 14[5]
% female	81%	84%	81%	61%[1]	82%[3]	65%[5]
DHI	68.7 ± 14.3	72.0 ± 13.6	69.0 ± 13.5	~30-70[2]	36 ± 0.9[3]	23 ± 0.8[3]
HADS anxiety	11.1 ± 3.8	11.0 ± 3.9	9.4 ± 3.1	7.8 ± 4.2 [1]	7.1 ± 3.3[4]	6.2 ± 4.46[6]
HADS depression	8.4 ± 3.8	8.8 ± 3.2	7.0 ± 4.0	6.5 ± 4.1[1]	6.0 ± 3.3[4]	4.1 ± 3.3[6]

1. N=305 patients with PPPD reported by Axer, Finn, Wassermann, Guntinas-Lichius, Klingner, and Witte. 'Multimodal Treatment of Persistent Postural–Perceptual Dizziness'. *Brain and Behavior* 10, no. 12 (2020): e01864. https://doi.org/10.1002/brb3.1864.
2. Approximate DHI score range for N=122 patients taken from Figure 4 in Zhang, Jiang, Tang, Liu, and Li. 'Older Patients with Persistent Postural-Perceptual Dizziness Exhibit Fewer Emotional Disorders and Lower Vertigo Scores'. *Scientific Reports* 12, no. 1 (2022): 11908. https://doi.org/10.1038/s41598-022-15987-w.
3. N=365 from Chari., Liu, Chung, and Rauch. 'Subjective Cognitive Symptoms and Dizziness Handicap Inventory (DHI) Performance in Patients With Vestibular Migraine and Menière's Disease'. *Otology & Neurotology* 42, no. 6 (2021): 883–89. https://doi.org/10.1097/MAO.0000000000003081.
4. N=74; Kim, Lee, and Heo. 'Prevalence and Contributing Factors of Anxiety and Depression in Patients with Vestibular Migraine'. *Ear, Nose & Throat Journal* 103, no. 5 (May 2024): 305–12. https://doi.org/10.1177/01455613231181219.
5. N=5508 MD cases; Bruderer, Saskia G., Daniel Bodmer, Nadja A. Stohler, Susan S. Jick, and Christoph R. Meier. 'Population-Based Study on the Epidemiology of Ménière's Disease'. *Audiology and Neurotology* 22, no. 2 (2017): 74–82. https://doi.org/10.1159/000475875.
6. N=122, Söderman, Bagger-Sjöbäck, Bergenius, and Langius. 'Factors Influencing Quality of Life in Patients with Ménière's Disease, Identified by a Multidimensional Approach:' *Otology & Neurotology* 23, no. 6 (2002): 941–48. https://doi.org/10.1097/00129492-200211000-00022. In a larger study of N=358 MD volunteers mean anxiety score can be estimated > 8.7 given severity category proportions (Kirby, et al. 2009 https://doi.org/10.1016/j.jpsychores.2008.05.027).

Figure S1, Indications of rehabilitation effects for participants who adhered (N=17) or did not adhere (N=20) to recommended playtime. These groups did not differ in mean baseline VVAS ($F(2,73)=0.44$), symptom duration ($F(2,73)=0.12$), and age ($F(2,67)=1.16$), or counts of reported diagnoses (see Table 1 in main text and S1 above). Plots are a simplification of the data provided in the correlation plots in Figure 4 of main text. VVAS scores reduced for participants that played for the recommended time, while no reduction was evident for participants who played less, or for the control group (two-way 2 (Time 1 and Time 2) x 3 (Control, Recommended playtime Intervention, Low playtime Intervention) mixed ANOVA with repeated measures, interaction $F(2,73) = 4.33$, $p = 0.017$). DHI and NPQ showed no group x time interaction ($F(2,73) = 1.2$; $F(2,69) = 0.2$). Both anxiety and depression scores decreased for the group that played the recommended amount of time, while no reduction was evident for participants who played less or for the control group ($F(2,70) = 3.5$, $p = 0.037$, $F(2,70) = 3.5$, $p = 0.036$). Error bars are SEM. Shaded areas indicate categories associated with each measure, where available (for VVAS and DHI, pink=severe, orange=moderate; for HADS, pink = clinically diagnosable, orange = borderline, green = normal).

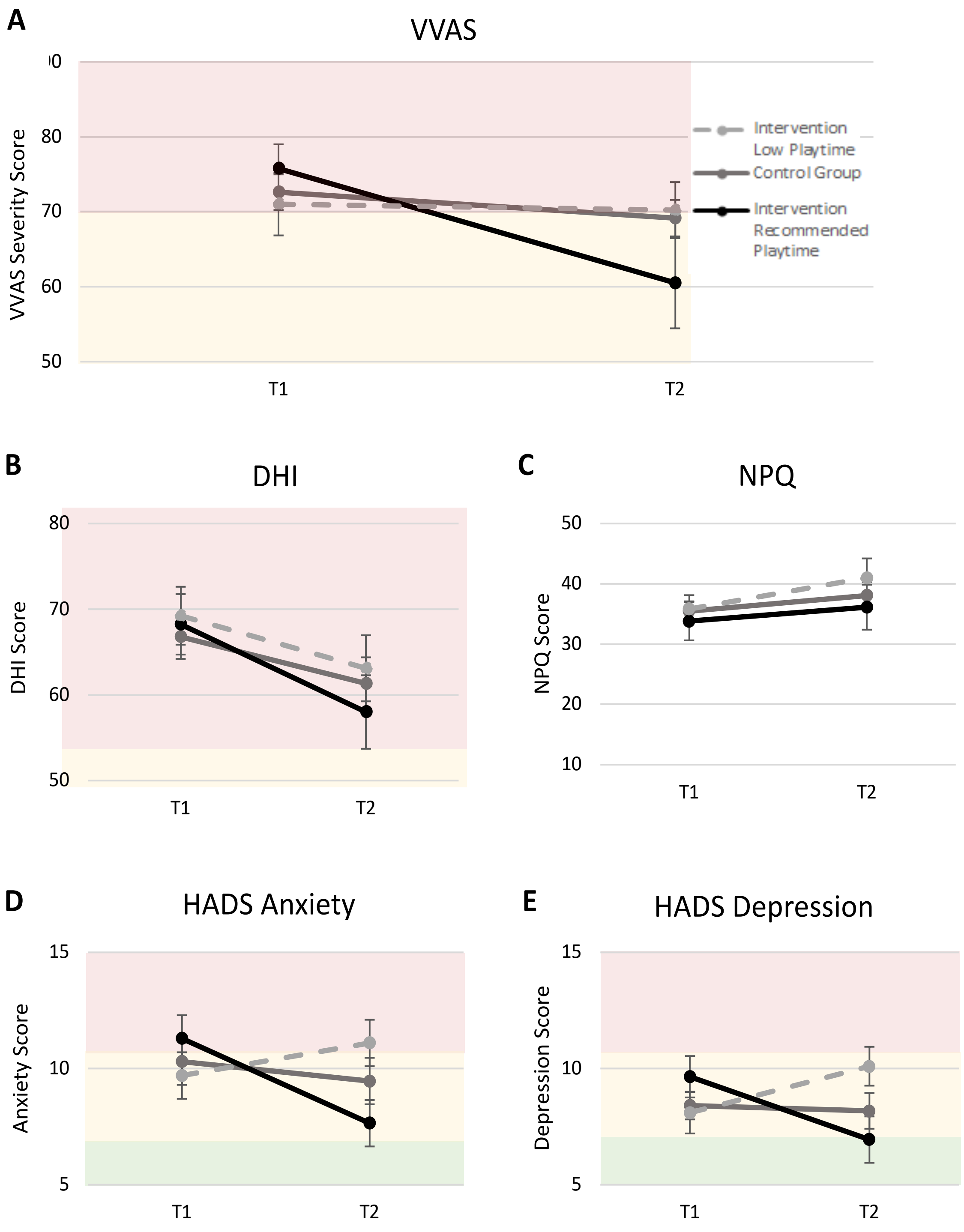

Supplement: Supplementary file 1 — Supplementary Information. [file 41598_2024_67745_MOESM1_ESM.pdf]
